# Improving methodology in heart rate variability analysis for the premature infants: Impact of the time length

**DOI:** 10.1371/journal.pone.0220692

**Published:** 2019-08-09

**Authors:** Trang Nguyen Phuc Thu, Alfredo I. Hernández, Nathalie Costet, Hugues Patural, Vincent Pichot, Guy Carrault, Alain Beuchée

**Affiliations:** 1 Laboratoire Traitement du Signal et de l’Image (LTSI – UMR 1099), Université de Rennes 1, Centre Hospitalier Universitaire de Rennes, Inserm, Rennes, France; 2 Pôle Mère-Enfants, Réanimation Néonatale – Hôpital Nord, Centre Hospitalier Universitaire Saint-Etienne, Saint-Etienne, France; 3 Système nerveux autonome - Epidémiologie Physiologie Ingénierie Santé (SNA-EPIS 4607), Université Jean Monnet, Saint-Etienne, France; Hopital Robert Debre, FRANCE

## Abstract

**Background:**

Heart rate variability (HRV) has been emerging in neonatal medicine. It may help for the early diagnosis of pathology and estimation of autonomous maturation. There is a lack of standardization and automation in the selection of the sequences to analyze and some features have not been explored in this specific population. The main objective of this study was to analyze the impact of the time length of the sequences on the estimation of linear and non-linear HRV features, including horizontal visibility graphs (HVG).

**Methods:**

HRV features were repeatedly measured with linear and non-linear methods on 2-, 5-, 10-minute sequences selected from the longest 15-min sequence and recorded on a weekly basis in 39 infants less than 31 weeks at birth. The associations between HRV measurements were analyzed through principal component analysis and k-means clustering. The effects of the time lengths on HRV measurements and post-menstrual age (PMA) were analyzed by linear mixed effect model for repeated measures.

**Results:**

The domains of analysis were concordant for their descriptive parameters of short (rMSSD, SD1 and HF) and long-term (SD, SD2 and LF) variability. *α*_1_ was correlated with the LF/HF and SD2/SD1. DC and AC were correlated with short-term variability estimates and significantly increased with GA and PMA. Shortening the windows of analysis increased the random measurement error for all the features and increased the bias for all but short term features and HVGs.

**Conclusion:**

The linear and non-linear measurements of HRV are correlated each other. Shortening the windows of analysis increased the random error for all the features and increased the bias for all but short term features and HVGs. Short-term HRV can be an index for evaluating the maturation in whatever sequence length.

## Introduction

Heart rate variability (HRV) analysis has been emerging as a promising diagnostic tool in neonatal care. Computer-based analysis of cardiac rhythms, using time and frequency domain analysis, entropy, scale invariance coefficient and Poincaré geometry, has proved useful in many settings. In this regard, specific heart period characteristics such as short deceleration, low entropy and decreased long-range fractal correlation have been associated with proven sepsis in premature infants [1, 2], viral infection [3], immunization [4, 5], pain [6, 7] and Kangaroo care [6, 8] in newborns.

HRV can be analyzed using very long recordings, i.e. 24 hours or more, to much shorter recordings, depending on the features of interest [9]. Analyzing HRV on very long-term recordings is difficult in premature infants because of their unstable clinical conditions, the various ventilation supports they need, the frequent changes in body position, the spontaneous movements and routine care that cause many artifacts. Thus, shorter recordings seem more convenient for clinical applications in premature infants. Most of the studies in premature infants used short recordings, i.e. 5-10 minutes. However, to our knowledge, there is no study performing HRV analysis on ultra-short sequences in premature infants, evaluating how the sequence length can affect HRV analysis.

Among the HRV features, some parameters can be extracted from a short recording, while others require longer data sets. Some researchers have investigated whether ultra-short sequences could replace the standard short-term sequences (at least 5 minutes). These studies were conducted on healthy adults and they pointed out that rMSSD is measurable from a record less or equal to 2 minutes [10, 11]. In most cases, this is a requirement related to the underlying mathematical assumptions of each method, whatever linear or non-linear methods. In this respect, horizontal visibility graphs (HVG), a new method of measuring HRV with different underlying principles [12, 13], has not been explored for its robustness to changes in time length.

Furthermore, HRV refers to the regulation of the sinoatrial node by the sympathetic and parasympathetic branches of the autonomic nervous system. It provides a window for observing the resulting beat-to-beat fluctuations in the rhythm of the heart in response to regulatory impulses. In light of this information, maturation aspects of autonomic regulation have been explored using HRV. In these studies, HRV was analyzed on long sequences, from 10 minutes [14, 15], 15 minutes [16] to 2,5 hours [17]. These studies aimed at quantifying long-term variability, the very long-range correlations and multi-fractal properties of heartbeat dynamics related to autonomic activity and its cascade feedback loops to control heart rate regulation [18]. In a similar way as above, the short-term autonomic regulation of heart rhythm or which is the shortest recording for analyzing autonomic nervous system has not been explored.

The main objective of this study was to analyze the effect of time length of the signal on the estimation of linear and non-linear HRV features, including horizontal visibility graphs (HVG). Firstly, we provided a comprehensive insight into the association between HRV features. Then we performed iterative measurements on 2-, 5-, 10- and 15- minute sequences to investigate the impact of these different time lengths on features estimating autonomic maturation in the preterm infants.

## Materials and methods

### Study population

The study was an ancillary of a longitudinal cohort study of premature infants conducted in the neonatal unit of the University Hospital of Saint-Etienne, France, from August 2004 to July 2005 [16], and it was approved by the Commission Nationale de l´Informatique et des Libertés (CNIL) and by the ethics committee of Saint-Etienne University Hospital in January 2004. The information of study was informed by explanatory leaflet with time for reflection and the consent was obtained by writing, with signature of parents or legal representatives.

Thirty-nine premature infants less than 32 weeks at birth and consecutively hospitalized in the intensive care unit were included in this study. The exclusion criteria were as follows: the presence of neurological or cardiac congenital defects; cardiac arrhythmia or the absence of sinus rhythm; need for prolonged resuscitation at birth and/or an Apgar score less than 5 at 5 min; and any maternal nicotine use during pregnancy. Two premature infants were 25 weeks, one was 26 weeks, five were 27 weeks, sixteen were 28 weeks, seven were 29 weeks, four were 30 weeks, and four were 31 weeks gestational age (GA). All of the premature newborns received a daily dose of caffeine citrate adjusted to the weekly plasma caffeine levels. None received morphine during hospitalization.

### Data collection

Each premature infant underwent at least 15-minute recordings once per week from the week of birth or following birth to 41 weeks post menstrual age (PMA) or discharge. All of the recordings were obtained during quiet sleep, identified through physiological and behavior monitoring [19], 30 minutes after a morning-time feeding period (between 8 and 12 a.m. without painful or stressful procedures for at least 6 hours). Recording was delayed by 48 hours when there was an unstable acute pathology at the scheduled time of recording or a thermoregulation disorder at the time of the examination or when general anesthesia or drugs with cardiac effects were administered in the 7 days preceding the recording. All of the infants were required to be supine during recordings. The environmental conditions were controlled for incubator and ambient temperatures as well as for child position. During the recordings, clinical status, events, medications and ventilation parameters were noted and recorded.

All of the infants were continuously monitored with an IntelliVue MP40 patient monitor (Phillips Medical System, Eindhoven, Netherlands) with a Multi-Measurement Server M3001A, a noninvasive blood pressure module, a FAST-SpO2 module and a Microstream CO2 Extension (M3015A—Oridion Medical, Ltd.), which provided continuous monitoring of heart rate (3 lead-ECG), respiratory rate, temperature, SpO2 and systolic, diastolic and mean arterial pressure. A dedicated computer was devoted to record these signals for later analysis. All data were fully anonymized.

### Signal processing

The acquired ECG signals were processed using a set of custom signal processing scripts designed in Matlab software (The Mathworks, Inc.) at the Laboratoire Traitement du Signal et de L’image (INSERM UMR1099, University of Rennes 1). A noise-robust QRS detection method was applied to obtain the RR series over the whole data acquisition period (typically 15 minutes). The obtained RR series were manually verified for detection issues and corrected when needed. The obtained series were finally cut on segments of different lengths for further processing [20] by linear and non-linear methods. These time lengths and methods are described here after.

#### Definition of time length

Time length of the sequences were defined as long (L) for the whole 15-minute sequences that was weekly recorded for each patient, short (S5 or S10) for 5 or 10-minute sequences and ultra-short (US) for the 2-minute sequences that were encompassed in above-mentioned at least 15-minute sequences. The ultra-short and short sequences were extracted consecutively with 50% overlap from the whole recording.

**Linear methods**
- Time domain analysis: It consisted of the extraction of the mean (Mean), the standard deviation (SD), which is an estimate of global variability, and the square root of the mean squared differences of successive samples of the series (rMSSD), which is an estimate of short-term beat-to-beat variability [9].
- Frequency domain analysis: RR series, re-sampled at 4 Hz, were also analyzed in the frequency domain by an auto-regressive estimation of the power spectrum of order 12 and by integration of the low-frequency (LF) (0.02-0.2 Hz) and high-frequency (HF) (0.2-2 Hz) spectral bands and the LF/HF. Very low frequency variations (0-0.02 Hz) were not considered for the analysis of these short-duration segments [21, 22]

**Non-linear methods**
- Poincaré plot analysis: In this method, a scatter plot of the current R-R interval against the preceding R-R interval. From the analysis of this plot, three indexes are obtained: the standard deviation of the instantaneous beat-to-beat RR interval variability (minor axis of the ellipse or SD1), the standard deviation of the continuous long-term RR interval variability (major axis of the ellipse or SD2) as well as the axis ratio (SD2/SD1) [23].
- Sample entropy (SampEn): This marker provides an estimation of the regularity of the selected RR series. A high value of entropy reflects a strongly irregular and unpredictable sequence, while a low value reflects abnormal oversimplification. SampEn was calculated with a fixed window length (m) and tolerance (r) parameters. These parameters were optimized according to the strategy proposed by [24] on the database and finally we selected m = 3 and r to be 0.25 that resulted in the best value of the efficiency metric, which was less than 0.15.
- Detrended Fluctuation Analysis (DFA): The long-range dependence, i.e., the scale invariance, was tested through Detrended Fluctuation Analysis (DFA). The DFA technique characterizes the RR series using a self-similarity parameter (*α*) that represents the long-range fractal correlation properties of the signal: *α* is 0.5 for white noise with uncorrelated randomness, 1 for 1/f noise and long-range fractal correlations, and 1.5 for Brown motion. We evaluated the fractal scaling exponent from 4 to 40 beats (*α*_1_), and from 40 beats to quarter length of the sequence (*α*_2_) [25].
- Deceleration capacity (DC) and acceleration capacity (AC) were also computed from the RR series by: *i*) detecting all of the intervals longer (for DC) or shorter (for AC) than the preceding interval; *ii*) defining a common temporal support for all deceleration or acceleration intervals; *iii*) applying phase-rectified signal averaging; and *iv*) quantifying DC and AC by the application of a four-term slope estimation. While DC is considered to reflect parasympathetic control of the sinus rhythm, the meaning of AC remains unclear [26].
- Horizontal visibility graphs (HVG, so called Z) were obtained by transformation of the RR series into graphs maintaining their inherent characteristics [12]. They reflect the fact that a sample of the RR series at time k is able to “see” another sample on the same time series. In this study, we used the topological property of HVG called HVG 4-node motifs (*Z*^4^) as previously described in [13].

### Statistical analysis

The distributions of HRV features were plotted and tested for normality using Q-Q plots and the Shapiro-Wilk test. Data were presented as median and quartiles (Q1, Q3). To explore associations among HRV features and to analyze simultaneously the large-scale behavior of the system, we performed a principal component analysis (PCA) including all the HRV features measured either on 2-, 5-, 10- or 15-min sequences. The squared coordinates of the variables were used to estimate the quality of their representation (cos^2^) and their contribution (cos^2^ of the variable * 100 / total cos^2^ of the component) to the first factorial plane. Then, to identify groups of variables, the features were classified through hierarchical and k-means clustering. The optimal number of clusters was determined by maximal average silhouette width. Finally, GA and PMA were projected on the first factorial planes as continuous supplementary variables to highlight correlation between maturation and HRV features.

The quality of estimation of the parameters was assessed on different time lengths by Median Absolute Deviation (MAD) from the median which is equal for of a selected parameter *X*^*j*^ to:

$$\begin{array}{c} MAD=median(|X_{i}^{j}-median\left(X^{j}\right)|) \end{array}$$ (1)

where median is the median value, $X_{i}^{j}$ is one realization of a particular HRV feature on a specific window (US, S5, S10, L) and $X^{j}=\{X_{1}^{j},X_{2}^{j},\ldots ,X_{n}^{j}\}$ is the vector of all the realizations of the particular HRV feature *X*^*j*^ on a specific window (US, S5, S10, L). Then, we analyzed the effects of the time length (2, 5, 10 and 15-minutes) of the sequences on the absolute measures and MAD of HRV features using linear mixed effects models. The patients were considered to be random effect, PMA and GA to be fixed effects. PMA (less than 28, 32, 36, more than 36 weeks) and sequences’ length (L, S10 S5 and US) were included as categorical variables. To re-mediate deviations from assumptions of the linear regression model, dependent variables with skewed distribution were transformed through the Box-Cox procedure. In addition, to calculate the mixed effects mean bias and limits of agreement (LoA), we analysed the differences of each time length to the mean value using a mixed effects regression model, including patients as a random effect and PMA and GA as fixed effects [27]. Absolute measures and MAD of the most representative variables of each cluster, i.e. with the highest cos^2^ value, were depicted against PMA and time length of the sequences in box plots. The statistical significance of the tests was set at 5%. Statistical analyses were performed using R software [28] with packages nlme [29], FactoMineR [30], factoextra [31] and cluster [32].

## Results

### Patient characteristics

The premature infants group consisted of 39 infants (25 boys and 14 girls) ranging from 25 weeks to 31 weeks at birth. Two premature infants were 25 weeks, one was 26 weeks, five were 27 weeks, sixteen were 28 weeks, seven were 29 weeks, four were 30 weeks, and four were 31 weeks gestational age (GA). Their mean birth weight was 1032 g, ranging from 504 g to 1750 g. Their 5 min Apgar scores ranged from 5 to 10. Some newborns did not complete the 10 successive weeks of recordings because of postnatal death (n = 1), early discharge to another neonatal center (n = 3) or early discharge to home (n = 18).

### Associations between HRV features

**Fig 1:**
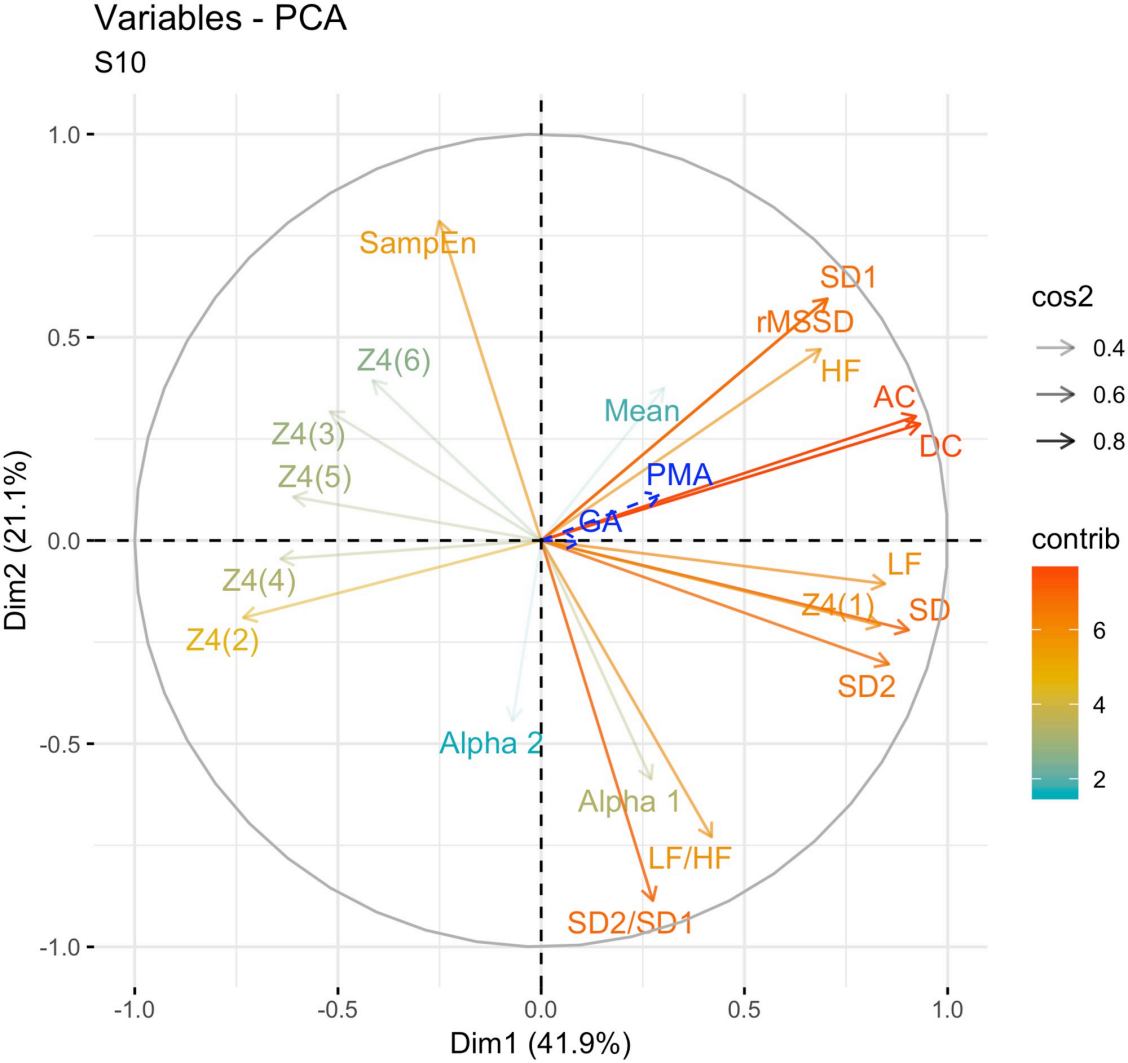
Principal component analysis of heart rate variability features estimated on 10-minute sequence. Projection of the variables on the factor map of the first 2 dimensions of the principal component analysis (63% of the variance). contrib: contribution of the variables calculated from their eigenvalues. cos2: squared coordinates of the variables, which is an estimate of the quality of their representation.

The principal component analysis performed on tables crossing all the features, showed similar projections on the first factor map whether the features were estimated on 2-, 5-, 10- or 15-min sequences, except for *α*_2_ that was poorly represented when estimated on 2- or 5-min sequences with a contribution below 1%. The first two dimensions explained more than 60% of the total inertia. As seen in Fig 1 where the features were estimated on 10-min sequences, the projection of the variables on the factor map showed good quality of representation and coherent associations for most of the features. *(i)* AC, DC, *(ii)* HF, rMSSD, SD1, *(iii)* LF, SD and SD2, *(iv)* LF/HF, SD2/SD1, *α*_1_, and *(v)* SampEn that appeared isolated. Nevertheless, squared coordinates of the variables Mean and *α*_2_ were low, indicating that they were poorly represented on this factor map of the first 2 components, whatever was the time length of the measurements. Indeed, they both mainly contributed to the third and fifth dimension. Similarly, all HVG features but $Z_{1}^{4}$ and $Z_{2}^{4}$ were poorly represented on the factor map of the first 2 dimensions. They mainly contributed to the fourth dimensions.

**Fig 2:**
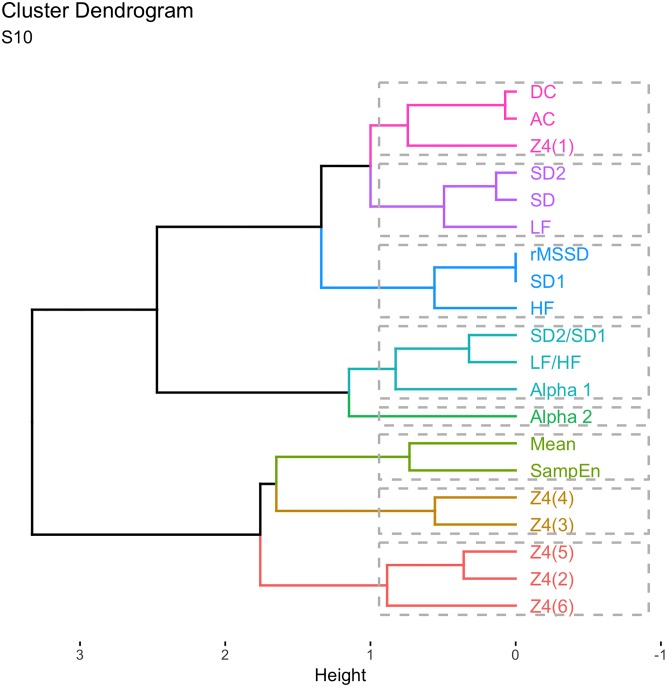
Dendrogram of the cluster analysis of HRV features estimated on 10-min sequences (S10). Each color shade represents a distinct cluster determined by the k-means clustering, which are the same clusters as determined by hierarchical cluster analysis (grey boxes).

The k-means clustering revealed lower variance within the clusters and higher variance between the clusters when the measurements were performed on 10-min sequences. The optimal number k of clusters was 8 and HRV features were allocated as follow: *(i)* $Z_{1}^{4}$, DC, AC; *(ii)* SD2, SD, LF; *(iii)* rMSSD, SD1, HF, *(iv)* SD2/SD1, LF/HF, *α*_1_ *(v)* *α*_2_ *(vi)* Mean, SampEn; *(vii)* $Z_{4}^{4}$, $Z_{3}^{4}$; and finally *(viii)* $Z_{5}^{4}$, $Z_{2}^{4}$, $Z_{6}^{4}$ (Fig 2). The greater distance we observed was between the group encompassing Mean, SampEn and all HVGs but $Z_{1}^{4}$ and the group of all the other features(Fig 2).

### Effect of the length of the sequences

**Fig 3:**
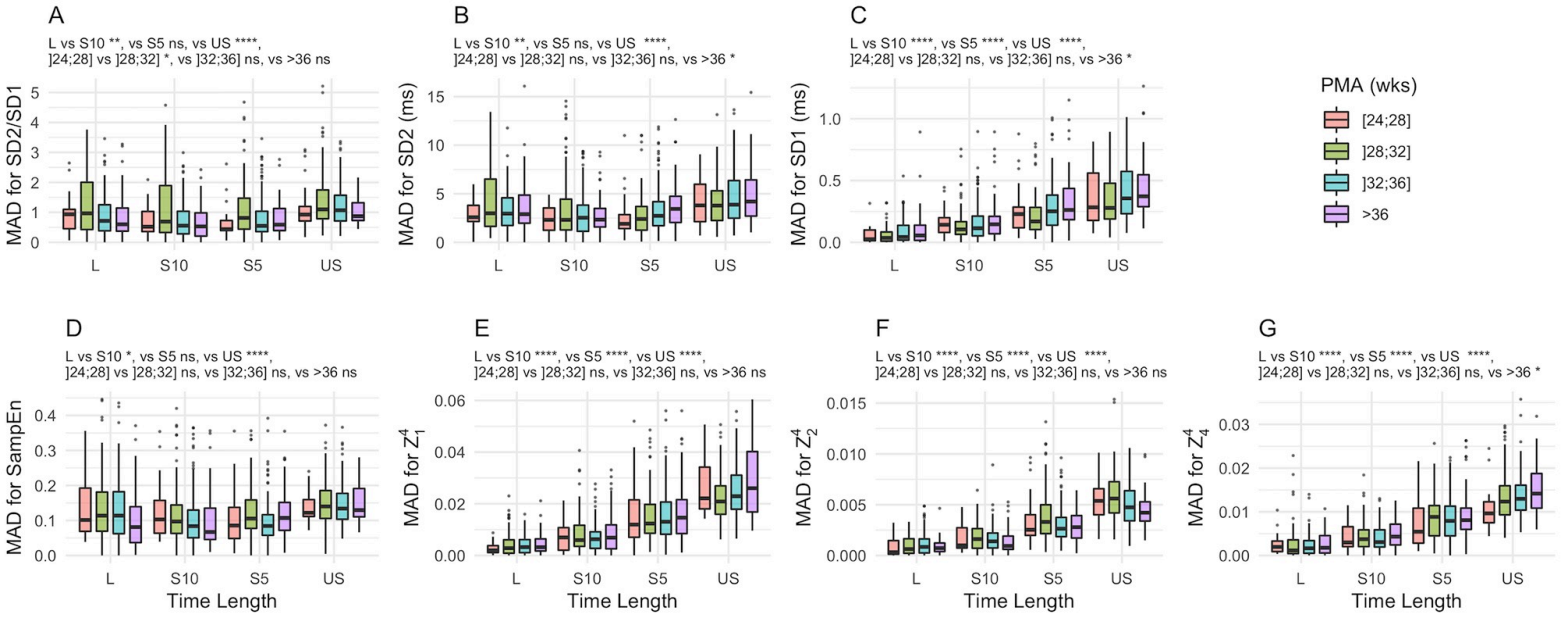
Median absolute deviation from the median of HRV features against time length of the sequence. Each box-plot represents median, IQR and extrema of Median Absolute Deviation of HRV feature measurement for a given time length against PMA. Color shades are respectively for PMA less than 28 wks (red), 28 to 32 wks (green), 32 to 36 wks (blue) and 36 wks and above (purple). The effects of time length (2-min (US), 5-min (S5), 10-min (S10) and 15-min (L)) were analyzed using linear mixed effects models p<0.05 (*), p<0.01 (**), p<0.001 (***) and p<0.0001 (****).

The time length of the sequences had a significant effect on the precision of the measurements of all the HRV features as determined by their absolute deviation from the median (Fig 3). These random measurement errors were greater for shorter time lengths. MAD was minimal for 10-min (S10) and 15-min (L) sequences, depending on the feature (Fig 3).

**Fig 4:**
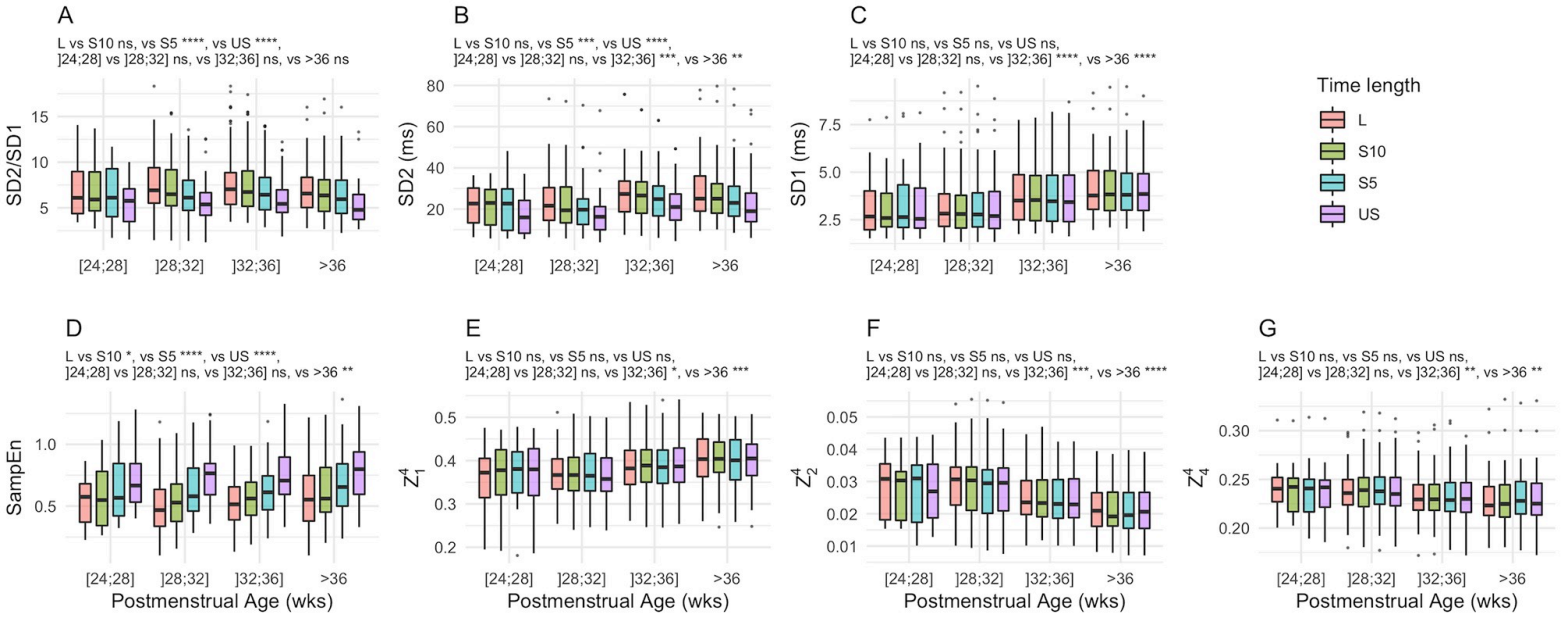
Absolute measurements of HRV features against PMA. Each box-plot represents an HRV estimate. The colors shades represent the different time lengths: 2-min (US), 5-min (S5), 10-min (S10) and 15-min (L). The effects of time length were analyzed using linear mixed effects models p<0.05 (*), p<0.01 (**), p<0.001 (***) and p<0.0001 (****).

Similarly, time length of the sequences had a significant effect on the accuracy of the measurements of most of the features. SampEn was significantly higher when measured on US (Fig 4D) and its mean bias was +21% [-10.6, 52.5]. SD2 and SD2/SD1 were significantly lower (Fig 4A and 4B). Their mean bias and LoA were -15.8% [-36.3, 3.8] and -13.8% [-30.5, 2.8] respectively. Besides, short-term features such as SD1 (Fig 4C) and HVGs (Fig 4E, 4F and 4G) had very low bias. Mean bias and LoA for SD1 ranged from 0.9% [-4.6, 6.3] to -1.1% [-6.8, 4.6] when measured on L and US sequences respectively. Mean bias for HVG was between -1.5 and +1.7%, minimal lower LoA -14% and maximal upper LoA was +12%.

### Effect of PMA on HRV features

Short-term HRV features significantly increased with PMA as seen in the PCA factor map of the first 2 dimensions (Fig 1) and in the representation of the absolute measurements of SD1 against PMA (Fig 4). Similarly, $Z_{2}^{4}$ significantly decreased with PMA. On the contrary, SD2/SD1 and LF/HF ratios, *α*_1_ and SampEn did not change significantly with PMA, whatever the sequence length.

## Discussion

This is the first study that extensively explore the maturation of linear and non-linear properties of HRV features in the preterm infants, including horizontal visibility graphs. The three main methods of HRV analysis were concordant for their descriptive features of short-term (rMSSD, SD1 and HF) and long-term (SD, SD2 and LF) variability. They increased with PMA, as did DC and AC. On the contrary, SD2/SD1 and LF/HF ratios, *α*_1_ and SampEn didn’t change with PMA. HVG and *α*_2_ seemed to carry distinct information. At last, shortening the time length of analysis increased random measurement error for all the features and systematic measurement error for all but short term features and HVGs.

### Impact of sequences’ length on HRV features

Effort to shorten the duration of recording is important to improve efficiency in both clinical and research settings. This is particularly true when analyzing HRV in premature infants that are hospitalized in intensive care units where sources of artifacts are frequent. Even if 24-hour HRV appears to be a gold standard for clinical HRV assessment since it encompasses many long term influences, e.g. core body temperature, metabolism, sleep cycles, and the renin–angiotensin system, a shorter recording may also be efficient in evaluating the relationship between sympathetic and parasympathetic nervous system at given time. Short-term HRV has been widely used for many years but there is no consensus on the time length for the measurements. Therefore, we aimed to explore the impact of sequences’ time length on HRV measurements comparing the 15-min time length used in the original study of Patural *et al*. [16] with shorter time lengths, i.e. ultra-short (2-min) and short (5-, 10-min) sequences.

The study of Shaffer *et al*. suggested the minimum duration that HRV could be calculated, for example: a 10-second segment successfully estimated mean heart rate; A 60-second segment estimated SDNN, rMSSD; a 90-second segment estimated, LF power, SD1, and SD2; a 120-second segment estimated DFA *α*_1_; a 180-second segment estimated, LFnu, HF power, HFnu, LF/HF power, SampEn, DFA *α*_2_; a 240-second segment estimated SampEn. [11]. In the current study, ultra short sequences were investigated for the very first time in the premature infants, a challenging subject exposed to many unstable clinical conditions and difficult artifacting procedures. We found that shortening the time length increased random measurement error and bias. Shaffer *et al*. only used the Pearson product-moment correlation coefficient that cannot account for random and systematic difference between the measurements. The method we choose allowed to detect such errors. On another side, our results are concordant with those of Munoz *et al*. They investigated to what extent (ultra-)short recordings capture the “actual” HRV on recordings of 10s, 30s, and 120s selected from the longest (gold-standard) recording of 240s to 300s. They found out that decrease in the bias and in the width of the 95%LoA interval were observed as the recording length increased from 10s to 120s, for both SDNN and rMSSD [10]. Indeed, we observed similar results for all HRV features when recording length increased from 120s to 600s.

### Interactions between linear and non-linear explanatory variables of the HRV

The correlations between HRV features have physiological origins above and beyond their mathematical relationships. The HF component of HRV is mainly under parasympathetic regulation, as observed in clinical and experimental observations of autonomic maneuvers, such as electrical vagal stimulation [33], muscarinic receptor blockade [34], and vagotomy [35]. Although they can also be influenced by the sympathetic nervous system [35], HF, SD1 and rMSSD are strongly affected by vagal activity [36]. The interpretation of the LF component is more complex. It has long been considered a marker of sympathetic modulation, especially when expressed in normalized units [37, 38] or as the LF/HF [35]. Nevertheless, sympathetic blockade does not suppress LF oscillations, while parasympathetic blockade strongly affects them, and sympathetically induced LF oscillations of blood pressure induce LF oscillations in the vagal outflow and RR through the baroreceptor reflex [36]. Consequently, the LF component appears to include both sympathetic and vagal influences. The relationship between AC, DC and spectral indices of HRV has observed in the study of Wang *et al*. As in our study, they pointed out a strong correlation between AC, DC and rMSSD, SD, LHF (low and high frequency) [39].

#### Poincaré plot geometry

The Poincaré plots, in which RR interval is plotted as a function of the previous RR interval, portray the nature of RR interval fluctuations. Poincaré plot analysis is both a quantitative and visual technique. In addition to the traditional linear methods that completely ignore the time series structure, Poincaré plot analysis can provide some additional information about the balance between short- and long-term variability. Poincaré plot analysis is easier and more sensitive at evaluating the sympatho-vagal balance and observing the beat-to-beat HRV. The very strong correlation between SD1 and rMSSD was described by Brennan *et al*. [40], then Hoshi *et al*. [23]. These two features have been shown both empirically and mathematically to be identical HRV metrics [41]. Correlations between SD, SD2 and LF on one side, and SD2/SD1 and DFA on the other, were also observed in the study of Hoshi *et al*. [23].

#### Entropy

Entropy estimation appears to be useful and may be calculated from much shorter series. Common estimates for calculating entropy rates in physiologic signals are SampEn and approximate entropy. In current study, we concentrated on the calculation of SampEn because this measure of complexity is less biased and more reliable [42]. It is assumed that SampEn depends on autonomic nervous system activity, but also on other mechanisms such as mechano-receptors afferent activity for example [43]. This could be the reason why SampEn mainly contributes to different dimensions while traditional HRV features mainly contribute to first and second dimensions.

#### Horizontal visibility graph

Horizontal visibility graphs are networks constructed of time series. Some recent researches proved benefits of using visibility graph for EEG analyses. In analyzing HRV, this method is still new and needs further investigation. HVG has been explored in some diseases in adults such as congestive heart failure or primary cardiomyopathy [44]. There is currently no study in the preterm infants. In the current study, one of the HVG features appeared to correlate with traditional HRV features, i.e. $Z_{1}^{4}$, while others had little similarities with time and frequency domain HRV features. Therefore, HVG appears to hold complementary information to traditional features and could be useful for HRV analyses.

### Clinical relevance

HRV analysis is a promising diagnostic tool in the neonatal care. Beside other physiological signs, HRV features add important information on the status of premature infants and may improve the performance of a decision support system. Our study pointed out that none of the features were robust to the changes in time length. The shortening of the signal was associated with an increased random measurement error in all features estimations and an increased bias in all features estimation but HF, rMSSD, SD1, AC, DC and HVGs. 10-min time length provided a good compromise with minimal error for all the features and minimal bias for SD2/SD1, SD2 and SampEn.

Many studies used frequency domain analysis to estimate autonomic maturation in preterm infants. They also used various time lengths to estimate HRV features, from 10 minutes [14, 15] or 15 minutes [16] to 2.5 hours [17]. As observed in the current work, the frequency domain feature HF is strongly correlated with rMSSD, SD1, AC and DC. All these short-term features significantly increase with PMA with minimal bias in their measurement when shortening the time length of the sequences. They consequently are better candidates for the estimation of autonomic maturation than SD2/SD1, LF/HF, *α*_2_ that do not significantly change with the PMA.

## Conclusion

Computer-based systems for the analysis of cardiac rhythm are very useful for diagnosis and disease management. Several projects intend to propose decision support system to assist clinicians in their decision-making that integrate physiological features. In this sense, this work can be seen as a new contribution in the difficult context of prematurity where signals are highly corrupted by noise and artifacts. The current study suggest that time length for the measurements has to be optimized and selected regarding regarding the question to explore. Comparisons have to done on features measured on the same time length. HRV features can be extracted from ultra-short sequences but with greater random and systematic errors.

## Supporting information

S1 Table Table of absolute measurements of HRV features. (CSV)

S1 Table Table of median absolute deviation from the median of HRV features. (CSV)
